# A Phase Ib/II Study of BXCL501 in Agitation Associated with Dementia

**DOI:** 10.1002/alz.089463

**Published:** 2025-01-09

**Authors:** Robert Risinger, Lavanya Rajachandran, Heather Robinson

**Affiliations:** ^1^ Bioxceltherapeutics, New Haven, CT USA; ^2^ Bioxcel Therapeutics, New Haven, CT USA; ^3^ BioXcel Therapeutics, New Haven, CT USA

## Abstract

**Background:**

BXCL501, a sublingual film formulation of dexmedetomidine, a highly selective α2 adrenoceptor agonist, is currently being studied for the acute treatment of agitation associated with dementia.

**Method:**

This was a Phase 1b/2 study assessing efficacy and tolerability of BXCL501 for treatment of acute agitation associated with dementia. Subjects were randomized to active treatment with BXCL501 (30, 40, or 60µg) or placebo. The main efficacy measure was PANSS Excited Component (PEC) score.

**Result:**

A total of 100 patients were enrolled in the study. Most subjects had moderate agitation at baseline based on the PEC score.

At 2 hours post‐dose (primary endpoint), a significant improvement from baseline in PEC total score was observed in the 60µg group compared with placebo (LS mean difference: ‐4.2, *P* = 0.0011). Significant improvements from baseline in PEC total scores were also observed at 1, 4, and 8 hours. In the 40µg group, significant improvements from baseline in PEC total score compared with placebo were observed at 1, 2, and 4 hours. No significant differences from placebo were observed in the 30µg group.

BXCL501 doses of 30, 40, and 60 µg were well tolerated in this patient population. None of the observed TEAEs were severe and no subjects discontinued the study due to an AE. The most frequently reported TEAE in the BXCL501 30, 40, and 60 µg treatment groups was somnolence (56.3%, 34.8%, and 60.0%, respectively); the incidence was 5.4% with placebo. Other adverse events of interest were hypotension (2 subjects each in 40µg and 60µg groups), orthostatic hypotension (one patient each in the 30 µg and 60 µg groups) and bradycardia (one patient in the 60 µg group). No cases of syncope or falls were reported in any of the groups.

**Conclusion:**

There are currently no FDA‐approved treatments for acute management of agitation in elderly patients with dementia. In this study, BXCL501 60 µg and 40 µg significantly reduced the symptoms of agitation at 2 hours after administration in this patient population, as measured by PEC. At those doses, the treatments were relatively well tolerated.

